# Anterolateral augmentation procedures during anterior cruciate ligament reconstructions in skeletally immature patients: Scoping review of surgical techniques and outcomes

**DOI:** 10.1002/jeo2.12012

**Published:** 2024-03-06

**Authors:** Martijn Dietvorst, Stéphanie Verhagen, Marieke C. van der Steen, Florens Q. M. P. van Douveren, Rob P. A. Janssen

**Affiliations:** ^1^ Department of Orthopaedic Surgery & Trauma Maxima Medical Centre Eindhoven The Netherlands; ^2^ Department of Orthopaedic Surgery & Trauma Catharina Hospital Eindhoven Eindhoven The Netherlands; ^3^ Orthopaedic Biomechanics, Department of Biomedical Engineering Eindhoven University of Technology Eindhoven The Netherlands; ^4^ Chair Value‑Based Health Care, Department of Paramedical Sciences Fontys University of Applied Sciences Eindhoven The Netherlands

**Keywords:** anterior cruciate ligament, anterolateral ligament reconstruction, lateral extra‐articular tenodesis, open growth plates, skeletally immatures

## Abstract

**Purpose:**

Graft failure rates after anterior cruciate ligament reconstruction (ACLR) in children and adolescents are higher compared to adults. Anterolateral augmentation procedures have recently generated increased focus regarding their ability to reduce graft failure rates. Concerns in skeletally immatures are potential growth disturbances and overconstraint after anterolateral augmentation. The aim of this scoping review is to provide an overview of all current anterolateral augmentation procedures in skeletally immature patients and to discuss surgical techniques, clinical and biomechanical outcomes.

**Methods:**

This scoping review was performed following the PRISMA (Preferred Reporting Items for Systematic Reviews and Meta‐Analysis) statement extension for scoping reviews. On 22 December 2022, an information specialist performed a systematic literature search in Cochrane, PubMed (Medline) and EMBASE databases. Inclusion criteria were anterolateral augmentation procedures, including lateral extra‐articular tenodesis (LET) and anterolateral ligament reconstruction (ALLR), in combination with ACLR in skeletally immatures.

**Results:**

Twenty studies were included after screening 1.485 abstracts. Seventeen studies describe LET techniques, four studies ALLR techniques and one study both techniques. Biomechanical data is scarce and shows conflicting results. Two studies compared ACLR with LET to ACLR in skeletally immatures with promising results in favour of the combined procedure. There were no differences in outcomes between LET and ALLR.

**Conclusions:**

Several LET and ALLR techniques have been described for skeletally immature patients and the first clinical data on LET and ALLR is available, which showed promising results. Further research is necessary to evaluate the risk of growth disturbances and overconstraint in skeletally immatures.

**Level of Evidence:**

Level IV.

**Abbreviations**
ACL: anterior cruciate ligament
ACLR: anterior cruciate ligament reconstruction
ACL‐RSI: Anterior Cruciate Ligament Return to Sport after Injury scale
ALL: anterolateral ligament
ALLR: anterolateral ligament reconstruction
G: gracilis
HS: hamstring
HSS Pedi‐FABS: Hospital for Special Surgery Pediatric Functional Activity Brief scale
ITB: iliotibial tract
KiRA: Kinematic Rapid Assessment
KOOS: Knee Injury and Osteoarthritis Outcome Score
LET: lateral extra‐articular tenodesis ST
LLD: leg length discrepancies
LSI: Limb Symmetry Index
n.s.: not significant
Pedi‐IKDC: Pediatric International Knee Documentation Committee
SANE: Single Assessment Numeric Evaluation
SD: standard deviation
ST: semitendinosus
STG: semitendinosus‐gracilis

## INTRODUCTION

Graft failure rates after anterior cruciate ligament (ACL) reconstruction in children and adolescents are higher compared to adults (13% vs. 5%) [1, 2, 11, 20, 41, 49]. Studies have highlighted young age, hyperlaxity, rotatory instability, concomitant injuries, sports participation and knee morphology as risk factors for graft failure [3, 15, 46]. High‐risk patients may benefit from anterolateral augmentation procedures during ACL reconstruction (ACLR) [45]. Anterolateral augmentation procedures have recently generated increased focus regarding their ability to augment an ACLR to reduce graft failure rates [4, 13, 15, 33, 39]. These procedures can be divided into lateral extra‐articular tenodesis (LET) and anterolateral ligament reconstruction (ALLR) techniques [33, 38]. Both LET and ALLR aim to prevent residual anterolateral rotary instability of the knee, which has been shown to decrease tension on the ACLR graft [10].

The literature on ALLR or LET in skeletally immature children and adolescents is scarce [4, 36]. This might be due to the fear of inducing growth disturbances by performing an anterolateral augmentation procedure, which makes this a controversial topic with paediatric ACL. In a systematic review, Carrozzo et al. [4] evaluated outcomes of over‐the‐top techniques, which includes a LET in its techniques, in skeletally immature patients and found low graft failure, low growth disturbance and high return to sport rates [4]. In addition to these LET techniques, several other anterolateral augmentation surgical techniques have been developed for transphyseal, all‐epiphyseal or hybrid ACLRs [8, 36, 42]. There is no current overview of these techniques and their outcomes. The aim of this scoping review is to provide an overview of all current anterolateral augmentation procedures, both additional to the intra‐articular ACLR and as a continuation of the ACLR itself, in skeletally immature patients and to discuss biomechanical outcomes, surgical techniques and clinical outcomes.

## METHODS

This scoping review was performed following the PRISMA (Preferred Reporting Items for Systematic Reviews and Meta‐Analysis) statement extension for scoping reviews [48]. The general purpose for inducting a scoping review is to identify and map the available evidence and not to produce a critically appraised and synthesised answer to a specific question [32].

### Selection criteria

Table 1 presents the inclusion and exclusion criteria applied to identify relevant articles. In cadaveric studies, both skeletally immatures and matures were eligible for inclusion due to ethical considerations and as not the effects on growth can be measured in cadavers, but the focus was on biomechanical outcomes.

**Table 1:** Inclusion and exclusion criteria.

	Inclusion criteria	Exclusion criteria
Participants	Skeletally immature children/adolescents undergoing transphyseal, all‐epiphyseal, extraphyseal or hybrid ACLR Cadavers (independent of skeletal maturity) undergoing transphyseal, all‐epiphyseal, extraphyseal or hybrid ACLR for biomechanical testing	
Surgery	Additional anterolateral augmentation procedures, including LET or ALLR	
ACLR	Considered ACLR techniques – Transphyseal – Over‐the‐top/extraphyseal – All‐epiphyseal – Hybrid	Adult ACLR in closed physes
Parameters and outcomes	Surgical technique and considerations – Graft type – Fixation method and location – Tensioning – Relation with physes	
	Clinical outcomes – Clinical stability – Re‐rupture rates – Growth disturbances – Secondary injuries to meniscus or cartilage – Osteoarthritis	
	Biomechanical characteristics	
Study design	Randomised controlled trial Cohort studies Case–control studies Case series or reports Technical notes Biomechanical studies	Systematic review Scoping review Narrative review
Language	English, Dutch	Other languages

Abbreviations: ACLR, anterior cruciate ligament reconstruction; ALLR, anterolateral ligament reconstruction; LET, lateral extra‐articular tenodesis.

### Intervention

LET or ALLR techniques combined with a transphyseal ACLR, over‐the‐top ACLR, all‐epiphyseal ACLR or hybrid ACLR (partial epiphyseal and transphyseal) were included in this review. LET essentially involves fixing the iliotibial tract (ITB) to the femur and exists in many different techniques, including (modified) Lemaire technique, Losee technique, MacIntosh reconstruction and Ellison distal ITB transfer [7, 19, 26, 29]. In an ALLR, a ‘free graft’ is used as graft for the ALL to restore the anatomy of the ALL [5, 6].

### Search strategy

On 22 December 2022, an information specialist (E. D.) performed a systematic literature search in Cochrane, PubMed (Medline) and EMBASE databases, as shown in Appendix S1. All published articles up to 22 December 2022 were considered eligible. The following terms, including synonyms and closely related words, were used as index terms or free‐text words: ‘anterior cruciate ligament reconstruction’, ‘transphyseal’, ‘all‐epiphyseal’, ‘over‐the‐top’, ‘lateral extra articular’ and ‘anterolateral ligament’.

### Study selection

Two researchers (M. D., S. V.) independently screened the abstracts for eligibility by using the Rayyan QCRI app (http://rayyan.qrci.org) [35]. A full‐text version of all eligible studies was independently reviewed by the same two researchers (M. D., S. V.). All references of these studies were screened for additional eligible articles. Any disagreement between the reviewers during the different screening phases was resolved through discussion.

### Data collection and charting process

Two authors (M. D., S. V.) independently charted all relevant data in a predefined Excel sheet. Data related to surgical technique, biomechanics and clinical outcome was gathered. Any disagreement about the interpretation of the results was resolved through discussion.

Specific parameters of the surgical technique used in each study were collected, including the femoral attachment, tibial attachment, graft type, fixation method, knee angle during fixation, graft tension at fixation and associated ligament reconstruction procedures, as well as biomechanical data if available. Findings from biomechanical studies of ALLR and LET were collected, including knee translation, rotational torque, kinematics and position of knee during testing, as well as other relevant reported results. Clinical outcomes, such as graft failure, growth disturbances and joint degeneration, were charted.

### Synthesis of results

Results were divided based on the anterolateral augmentation technique, in LET and ALLR. Surgical techniques are described as descriptive as well as schematic in figures. Clinical outcomes are presented by means of tables. Outcomes of biomechanical studies were discussed separately, as studies compared LET to ALLR techniques.

## RESULTS

### Search

**Figure 1:**
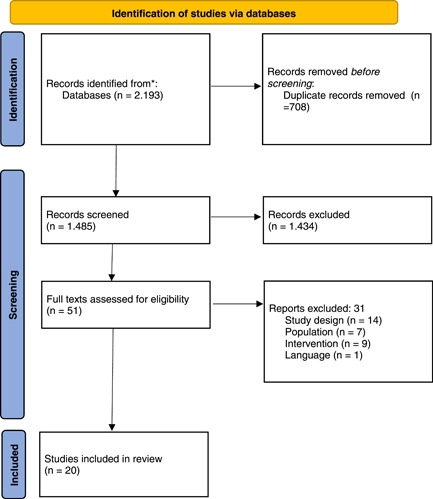
Flowchart of study selection.

Twenty studies were included in this scoping review after screening 1.485 abstracts (Figure 1). Seventeen studies describe LET techniques, four studies ALLR techniques and one study both techniques. In Appendix S2, the characteristics of the included studies are shown. Results are divided into the following topics: biomechanical outcomes, LET and ALLR.

### Biomechanical outcomes

Biomechanical data of ACLR with LET and ALLR are presented in respectively two and one cadaveric studies [21, 43, 47]. Types of ACLR and LET/ALLR with the most important results regarding stability and overconstraint are shown in Table 2. These studies showed different results on stability and overconstraint in comparable ACLR and anterolateral augmentation techniques. Overall evidence of biomechanical outcomes is low regarding paediatric anterolateral augmentation techniques, due to small numbers of cadaveric studies and cadaveric knees. ITB over‐the‐top ACLR with LET resulted in most studies in good stability and the addition of LET in the ITB over‐the‐top procedure improved rotational stability. Overconstraint in this technique is reported in two studies. All‐epiphyseal ACLR with LET or ALLR showed different results regarding restoring stability of the knee. Additional ALLR in all‐epiphyseal ACLR did not improve stability.

**Table 2:** Cadaveric studies evaluating biomechanical stability and overconstraint of different techniques.

Study	Experimental	Control	Results	
			Stability	Overconstraint
Kennedy et al. [21]	All‐epiphyseal hamstring ACLR and ITB over‐the‐top ACLR with and without LET techniques (*N* = 5)	ACL intact (*N* = 1)	AP translation significantly less in ITB over‐the‐top compared to other techniques	ITB over‐the‐top might overconstrain internal rotation and varus angulation movement
			Internal rotation is significantly less in ITB over‐the‐top compared to other techniques	
			All‐epiphyseal ACLR unable to restore rotational stability of ACL intact state, both with and without LET	
			ITB over‐the‐top had significantly less varus laxity in 45° and 60° of flexion compared to ACL intact state	
Sena et al. [43]	All‐epiphyseal hamstring ACLR and ITB over‐the‐top ACLR with and without LET techniques (*N* = 4)	ACL intact (*N* = 1) ACL deficient (*N* = 1)	All ACLR techniques restored the anterior displacement, internal rotation, posterior translation velocity and external rotational velocity to within ACL intact ranges	The ITB over‐the‐top ACLR (including LET) overconstrained anterior displacement by 38% and internal rotation by 52%
			The all‐epiphyseal ACLR technique (without LET) seemed most effective in restoring native knee kinematics under dynamic loading conditions that mimic the pivot shift test	
Trentacosta et al. [47]	All‐epiphyseal hamstring ACLR with or without ALLR and over‐the‐top ACLR with and without LET techniques (*N* = 4)	ACL/ALL intact (*N* = 1) ACL deficient ALL intact (*N* = 1) ACL/ALL deficient (*N* = 1)	ITB over‐the‐top ACLR including LET (ALL intact or deficient states) and all‐epiphyseal ACLR (ALLR state or ALL deficient state) were less stable in anterior displacement at all flexion angles (expect 0°) compared to the ACL intact state	None
			The all‐epiphyseal ACLR with ALLR and without ALL (transected) was less stable than the ITB over‐the‐top ACLR in anterior displacement at all angles except 90° of flexion	
			Addition of ALLR in the all‐epiphyseal ACLR did not improve rotational stability; addition of LET in the ITB over‐the‐top has a stabilising effect	
			Varus and valgus rotation was not significant between different ACLR techniques with and without ALLR	

Abbreviations: ACLR, anterior cruciate ligament reconstruction; ALLR, anterolateral ligament reconstruction; ITB, iliotibial band; LET, lateral extra‐articular tenodesis.

### LET

#### Techniques

**Figure 2:**
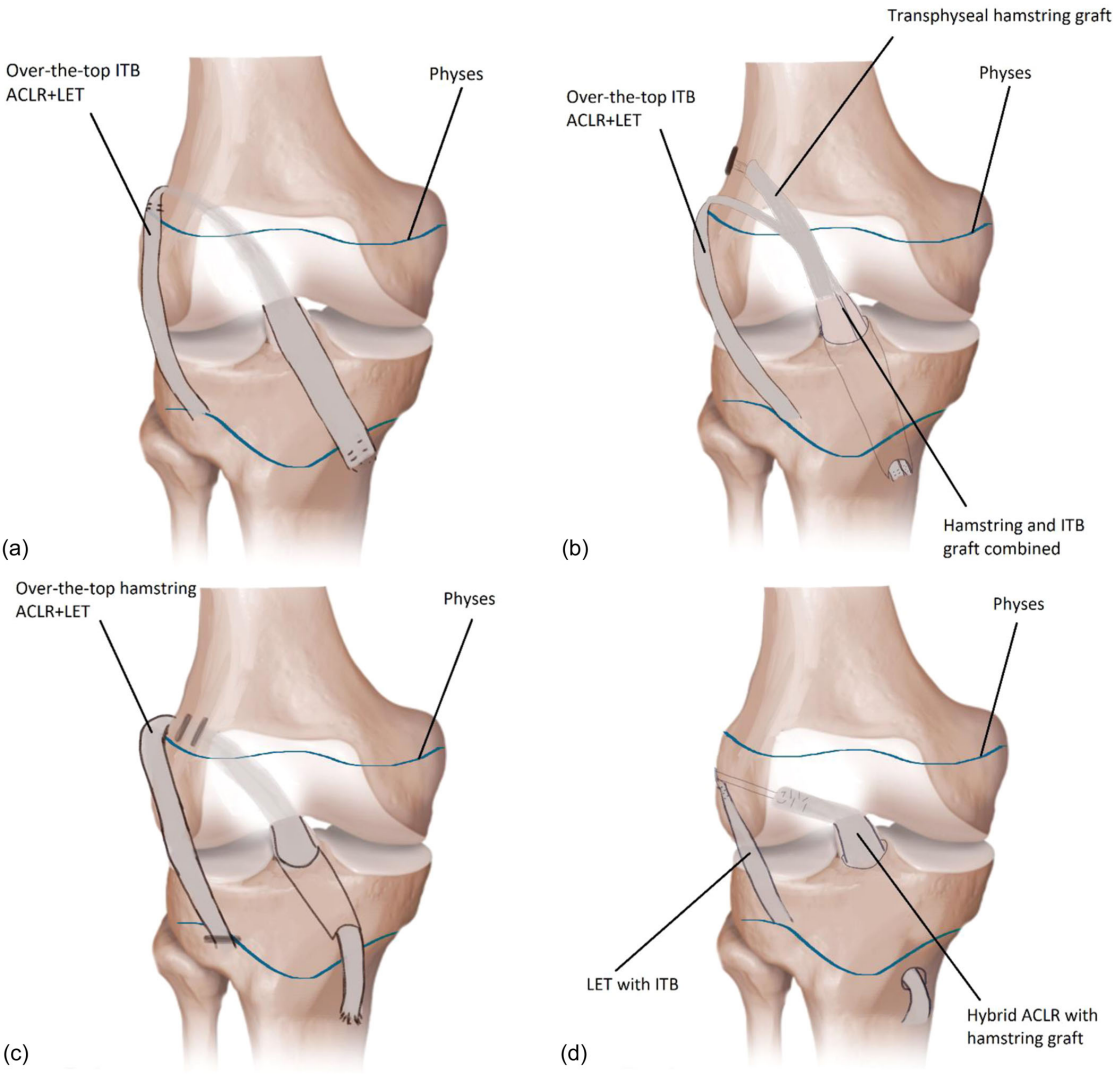
Different techniques of anterior cruciate ligament reconstruction (ACLR) in combination with lateral extra‐articular tenodesis (LET). (a) An iliotibial tract (ITB) over‐the‐top technique with LET [22, 23], (b) a hybrid ACLR with ITB LET [51], (c) an extra‐physeal hamstring over‐the‐top with LET [24, 40] and (d) a hybrid ACLR with hamstring tendons and ITB LET [14].

Seventeen studies described LET techniques in skeletally immature patients [8, 12, 14, 21, 22, 23, 24, 25, 30, 37, 40, 42, 43, 44, 47, 50] (Appendix 3). Nine techniques were based on the MacIntosh over‐the‐top technique [21, 22, 23, 30, 43, 44, 47, 50, 51], five on the Lemaire technique [8, 14, 25, 37, 42], two on Marcacci over‐the‐top technique [24, 40] and one on Ellison technique [12]. A schematic representation of the different ACLR techniques in combination with LET is presented in Figure 2. Of the MacIntosh over‐the‐top technique two alternatives have been used namely using the ITB (Figure 2a) or a combination of ITB and hamstring (HS) graft (Figure 2b). An example of a Marcacci technique is shown in Figure 2c and a Lemaire LET with hybrid ACLR in Figure 2d. As no description of the ACLR technique was provided in the manuscript, no example of the Ellison LET technique is shown in Figure 2 [12]. The Ellison LET technique involved a detachment of a strip of the ITB from Gerdy tubercle, reflection proximal to the lateral collateral ligament and fixation to Gerdy tubercle while passing the ITB strip under the lateral collateral ligament [12].

Fifteen studies used solely ITB as a graft for LET, and two studies used HS tendon autograft as LET graft [24, 40]. The specific locations of fixations of the grafts and fixations methods varied among ACLR techniques (see Appendix S3 for an overview). The LET was most commonly combined with an ACLR over‐the‐top technique [21, 22, 23, 24, 40, 43, 44, 47, 50, 51]. In two studies, a transphyseal ACLR was performed [8, 30]. A hybrid ACLR was described in five studies, of which Foissey et al. [14], Leyes‐Vence et al. [25] and Perelli et al. [37] performed an epiphyseal femoral tunnel and transphyseal tibial tunnel, and Shamrock et al. [44] and Wilson et al. [51] performed an over‐the‐top (and transphyseal by Wilson et al. [51]) femoral fixation and transphyseal tibial fixation. The ACLR technique was not specified in two studies [12, 42].

#### Clinical outcomes

Two studies [30, 37] compared the results of ACLR with LET with ACLR without LET, of which the results are shown in Table 3. Both studies showed significant better knee anterior–posterior and rotational stability and reduced graft failure rates in the ACLR + LET groups versus isolated ACLR [30, 37]. Rate of growth disturbances was low in both studies for ACLR and ACLR + LET and high PROMs and return to sports rates were presented [30, 37].

**Table 3:** Clinical outcomes of isolated ACLR versus ACLR + LET.

	Monaco et al. [30] (*N* = 111)			Perelli et al. [37] (*N* = 66)		
	Isolated ACLR (*N* = 40)	ACLR + LET (*N* = 71)	*p* Value	Isolated ACLR (*N* = 34)	ACLR + LET (*N* = 32)	*p* Value
*Characteristics*						
Mean age (years) (±SD)	16.3 (1.3)	16.1 (1.5)	0.471	13.5 (1.2)	13.8 (1.4)	0.792
Bone age, years (±SD)				14.0 (0.9)	14.1 (1.0)	0.897
Tanner stage, % (*n*)			0.127			
3/4	63 (25)	49 (35)				
5	38 (15)	51 (36)				
Mean follow‐up, months (SD)	36.5 (15.6)	47.9 (17.2)	**0.001**	26.6 (4.2)	25.1 (2.2)	0.591
*Outcomes*						
Laxity tests, % (*n*)						
KT‐1000 arthrometer (difference)			**<0.001**	1.9 (1.1)	0.8 (0.8)	**0.031**
0–2 mm	57 (20)	89 (54)				
3–5 mm	26 (9)	12 (7)				
>5 mm	17 (6)	0 (0)				
Pivot shift			**0.016**			
Grade 0	71 (25)	90 (55)				
Grade 1	11 (4)	25 (15)				
Grade 2	6 (2)	0 (0)				
Grade 3	11 (2)	0 (0)				
KiRA triaxial accelerometer (m/s^2^)				0.98 (1.12)	−0.59 (1.05)	**0.012**
Complications % (*n*)						
Graft failure	15 (6)	0 (0)	**0.003**	15 (5)	6 (2)	**0.021**
Contralateral ACL injury	3 (1)	0 (0)	0.284			
Growth disturbances	0 (0)	0 (0)	n.s.	3 (1)	3 (1)	n.s.
Infection	0 (0)	1 (1)	0.907			
Cyclops	0 (0)	1 (1)	0.907			
Joint degeneration				0 (0)	0 (0)	n.s.
PROMs scores, means (±SD)						
Overall KOOS	89 (8.2)	91 (8.1)	0.548			
Pedi‐IKDC				86 (8.4)	91 (9.6)	0.072
HSS Pedi‐FABS				18 (3.2)	19 (4.0)	0.180
Tegner, median	6	7	**0.010**			
Return to sport						
% (*n*)	85 (29)	92 (65)	0.523	82 (28)	91 (29)	0.059

*Note*: *p* Values are significant in bold.

Abbreviations: ACLR, anterior cruciate ligament reconstruction; HSS Pedi‐FABS, Hospital for Special Surgery Pediatric Functional Activity Brief Scale; KiRA, Kinematic Rapid Assessment; KOOS, Knee Injury and Osteoarthritis Outcome Score; LET, lateral extra‐articular tenodesis; n.s., not significant; Pedi‐IKDC, Pediatric International Knee Documentation Committee; SD, standard deviation.

In eight studies, noncomparative clinical outcomes of an ACLR with LET are described (Table 4) [12, 21, 23, 24, 30, 40, 44, 50, 51]. Mean age was ≤13 years in all studies except one study, which included both skeletally mature and immature patients [12]. Only one study presented additional bone age [51] and two studies Tanner stages [22, 40]. All studies had a mean follow‐up of ≥2 years. In most of these studies, additional LET resulted in relatively low graft failure rates (0%–17%), low growth disturbance rates (0%–17%) and high PROMs and return to sports rates.

**Table 4:** Clinical outcomes of LET.

	Feller et al. [12] (*n* = 25)^a^	Kocher et al. [22] (*n* = 237) (240 knees)	Lanzetti et al. [24] (*n* = 42)	Di Sarsina et al. [40] (*n* = 20)	Shamrock et al. [44] (*n* = 12)	Willimon et al. [50] (*n* = 12) (22 knees)	Wilson et al. [51] (*n* = 56) (57 knees)
*Characteristics*							
Mean age (years)	18.5 [range: 14–29]	11.2 (SD ±1.7)	12.5 [range: 11–14]	12.3 (SD ±1.7)	12.8 (SD ±1.8)	11.8 [range: 10–14]	13.0 [range: 11–16]
Bone age (years)							13.9 [range: 12–16]
Tanner stage, % (*n*)							
1/2		100 (237)		25 (5)			
3/4		0 (0)		20 (4)			
5		0 (0)		55 (11)			
(Mean) follow‐up	24 months	25.8 months	96.1 months	54 months [range: 34–123]	2.3 years [range: 1–5]	3 years [range: 1–7]	38.5 months [range: 24–78]
*Outcomes*							
Laxity tests, % (*n*)							
KT‐1000 arthrometer (difference)			Mean 1.2 mm [range: 0.9–1.5]				
0–2 mm	94 (17)			95 (19)			
3–5 mm	6 (1)			5 (1)			
6–10 mm	0 (0)			0 (0)			
>10 mm	0 (0)			0 (0)			
Pivot shift		*n* = 225					
Grade 0	91 (20)	99 (223)		0 (0)	100 (12)		
Grade 1	9 (2)	9 (2)		100 (20)	0 (0)		
Grade 2	0 (0)	0 (0)		0 (0)	0 (0)		
Grade 3	0 (0)	0 (0)		0 (0)	0 (0)		
Complications							
Growth disturbances, % (*n*)			5 (2)			0 (0)	16.7 (3)^b^
Valgus–varus			5 (2)	15 (3)			16.7 (3)^b^
Recurvatum			0 (0)	5 (1)			0 (0)^b^
LLD			0 (0)	0 (0)			5.5 (1)^b^
Reinjuries, % (*n*)		*n* = 137					
Ipsilateral	4 (1)	7 (9)	5 (2)	0 (0)	17 (2)	14 (3)	5 (3)
Contralateral	8 (2)	6 (13)	2 (1)	0 (0)	17 (2)		9 (5)
Infection, % (*n*)	4 (1)	0.4 (1)	2 (1)				
Cyclops, % (*n*)	4 (1)						
Joint degeneration, % (*n*)			0 (0)	10 (2)			5.4 (3)
PROMs scores	Means [range]	Means (SD) (*N* = 128)	Mean (range)	Medians [range]	Means [range]	Means (SD) (*N* = 19) (excl. graft failures)	
(Pedi‐)IKDC	92 [75–100]	93 (11)	95 (78–100)			97 (3)	91 (47–100)
KOOS	77 [57–100]						
Lysholm		93 (10)	95 (77–100)	99 [97–100]		95 (6)	
HSS Pedi‐FABS				100 [95–100]			22 (4–30)
ACL‐RSI	85 [61–100]						
SANE	94 [80–100]						
Tegner		8 [range: 2–10]			9 [7–10]	Median 8 [range: 6–10]	
Marx Activity scale	13 [6–16]			7 [3–9]			
Return to sport,^c^ % (*n*)	74 (17)	97 (124)	100 (42)	100 (20)		79 (15/19)	91 (52)

Abbreviations: ACL‐RSI, Anterior Cruciate Ligament Return to Sport after Injury scale; HSS Pedi‐FABS, Hospital for Special Surgery Pediatric Functional Activity Brief scale; KiRA, Kinematic Rapid Assessment; KOOS, Knee Injury and Osteoarthritis Outcome Score; LET, lateral extra‐articular tenodesis; LLD, leg length discrepancies; LSI, Limb Symmetry Index; n.s., not significant; Pedi‐IKDC, Pediatric International Knee Documentation Committee; SANE, Single Assessment Numeric Evaluation; SD, standard deviation.

^a^ Four skeletally immature patients.

^b^ Greater than 18 months remaining growth (*n* = 18).

^c^ Data available from 23 patients at 2 years follow‐up.

### ALLR

#### Techniques and outcomes

Four studies described ALLR techniques for skeletally immature patients [14, 31, 36, 47] (Appendix S4).

**Figure 3:**
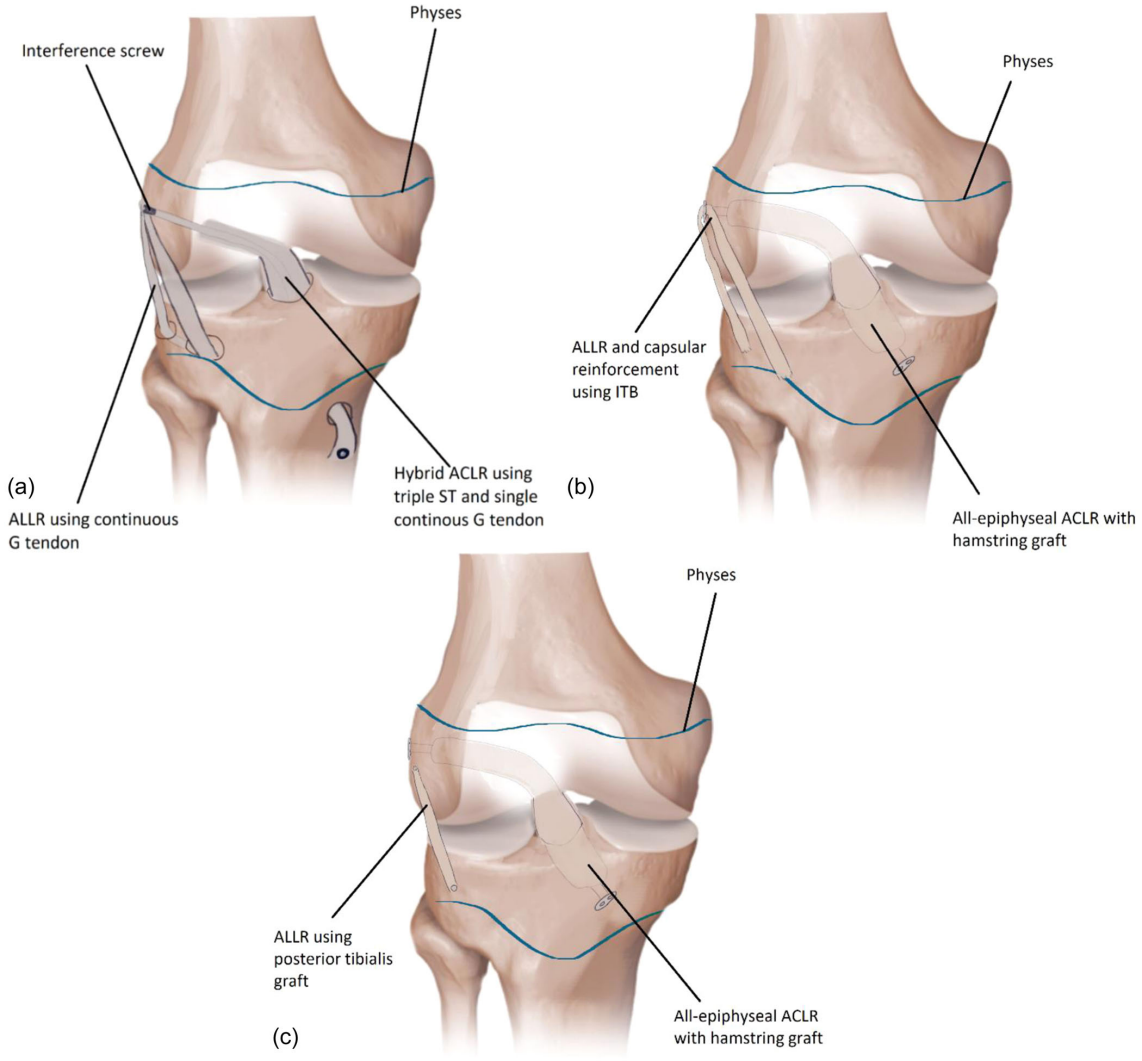
Anterolateral ligament reconstruction (ALLR) techniques. (a) Hybrid anterior cruciate ligament reconstruction (ACLR) technique using triple semitendinosus (ST) graft and single gracilis (G) graft, which is continuous as ALLR graft [14, 31]. (b) All‐epiphyseal hamstring ACLR using strip of ITB as ALLR and capsular reinforcement [36]. (c) All‐epiphyseal hamstring ACLR using posterior tibialis tendon as ALLR [47].

ACLR + ALLR described by Foissey et al. and Morin et al. were similar and a schematic representation of this technique is shown in Figure 3a [14, 31]. Both techniques were hybrid ACLR with a continuous HS graft, which is used as ALLR [14, 31]. The continuous graft consisted of a three‐strand semitendinosus and one‐strand gracilis tendon [31]. Two convergent, epiphyseal tunnels are created medial and lateral to Gerdy tubercle [31]. For the ACLR, a femoral all‐epihyseal and tibial transphyseal were made [31]. The ACLR graft is fixed in the femoral tunnel, and the ALL graft is passed in a posteroanterior fashion through the ALLR tibial tunnels under the iliotibial band and attached to the ACL traction wire at the femur in extension and neutral rotation [31].

Patel et al. [36] presented an ALLR technique, performed after a closed‐socket, all‐epiphyseal ACLR technique (Figure 3b) [36]. In this ALLR technique, the ITB was used for both anterolateral capsular reinforcement and ALLR, by leaving the tibial insertion of the ITB intact, attaching the ITB posterior to the femoral ACL graft button and fixing the ITB at the midpoint between Gerdy's tubercle and the fibular head [36]. The anterolateral capsular reinforcement was fixed with the knee in 90° of flexion and the ALLR in full extension [36].

Trentacosta et al. [47] presented an ALLR technique for skeletally immature patients in an adult cadaveric study [47]. The ALLR technique was performed by using a prepared posterior tibialis tendon allograft, which was fixed to femur and tibia in tunnels with an anchor (Figure 3c) [47]. The position of the tunnels was slightly proximal and posterior to the lateral epicondyle of the femur and 1 cm distal to the joint space on the midpoint between Gerdy's tubercle and the fibular head [47]. Graft tensioning was performed with the knee in extension [47].

#### Clinical outcomes

In contrast with studies LET clinical outcomes on ALLR are very scarce. Only Foissey et al. [14] reported complications after ACLR + ALLR in comparison to the outcomes of hybrid ACLR + LET (Table 5). Mean age of these patients was 13.8 (±1.4) years and all bone ages were <13.5 years in girls and <15.5 years in boys. There were no differences in outcomes between ACLR + LET versus ACLR + ALLR [14].

**Table 5:** Comparison of complications after ACLR + ALLR versus ACLR + LET by Foissey et al. [14].

Complications, *N* (%)	ACLR + ALLR (*n* = 19)	ACLR + LET (*n* = 20)
Graft failure	0 (0)	1 (5)
Meniscal suture failure	1 (5)	1 (5)
Arthrofibrosis	1 (5)	1 (5)
Growth disturbances	1 (5)^a^	1 (5)

Abbreviations: ACLR, anterior cruciate ligament reconstruction; ALLR, anterolateral ligament reconstruction; LET, lateral extra‐articular tenodesis.

^a^ Required contralateral epiphysiodesis because of overgrowth.

## DISCUSSION

The most important finding of this scoping review was that several LET and ALLR techniques have been described for skeletally immature patients and first clinical data on LET and ALLR is available. To this date, two studies compared ACLR with LET to ACLR in skeletally immature patients with promising results in favour of the combined procedure [30, 37].

The role of the anterolateral structures of the knee in contributing to anterolateral rotatory stability of the knee has gained interest in recent literature [12, 18, 27, 28]. In response to this, the Anterolateral Complex Consensus Group suggested some possible indications for a LET procedure as an augmentation of an ACLR [12, 16, 45]. These indications include: knee hyperextension greater than 10°, generalised hyperlaxity (Beighton score >4), grade 2 or higher pivot‐shift test result or revision ACLr [45]. Other risk factors for graft failure, such as age, sex, contralateral ACL injury and pivoting sport participation are taken into consideration for additional anterolateral augmentation [45]. Recent literature showed that additional anterolateral augmentation procedures help to reduce graft failure rates in young patients, who have the highest risk for graft failure [13, 15].

There is currently little evidence to guide surgical indications for anterolateral augmentation with concurrent ACLR in skeletally immature patients [36]. This population has an increased risk of graft failure because of their young age [2, 36]. Anterolateral augmentation during ACLR may therefore be considered to reduce graft failure rates in this population [4, 30]. Anterolateral augmentation procedures result however in additional surgical morbidity and can potentially result in overconstraint [21] and growth disturbances [40, 51]. Based on the currently available literature, graft failures rates might be lower in cases with anterolateral augmentation [30, 37]. It should be noted that evidence is still limited, especially on overconstraint and growth disturbances in this population.

The two studies that compared ACLR versus ACLR + LET showed promising results in favour of the combined procedure [30, 37]. In the study by Monaco et al. [30], no graft failures were found in the ACLR + LET group, which was significantly less than the 15% graft failure in the ACLR group [30]. Post‐ACLR laxity was also less in the ACLR + LET group. No differences in nongraft failure‐related reoperations or complications were found [30]. Perelli et al. [37] had similar findings of graft failure rates of 15% (isolated ACLR) versus 6% (ACLR + LET) and similar rates of growth disturbances between the two groups. The anterior–posterior and rotational stability was also significantly better in the ACLR + LET group [37]. Some of the noncomparative studies showed similar clinical results [12, 22, 23, 24, 40], although some studies found higher graft failure rates (14%–17%) [44, 50] and growth disturbance rates (17%) in patients with over‐the‐top LET [51]. Differences in the growth disturbance rates may be caused by the operative techniques, but also by the age of the patients and methods of determining growth disturbances. For example, Monaco et al. [30] included 16‐year‐old adolescents and evaluated growth disturbances clinically, whilst Wilson et al. [51] analysed adolescents with remaining growth of >18 months with lower limb radiographs [51].

To prevent these growth disturbances, all techniques described in this scoping review adjusted the anterolateral augmentation procedure to the physes. No sutures or staples were placed across open physes in any of the described techniques. Fixing the ITB proximal to the growth plate, however, could create a ‘tenodesis effect’ and possible growth disturbance, as it could act as a tether on the lateral side of the distal femoral physis and cause a valgus deformity [12, 25]. This phenomenon was demonstrated in a skeletally immature canine model when a transphyseal ACLR was excessively tensioned [9, 12]. Multiple studies fixed the LET proximal to the femoral growth plate, but only two of those studies evaluated growth disturbances [22, 23, 40, 50, 51]. Wilson et al. [51] found that the operated leg had greater valgus angle compared to the nonoperated leg in 16.7% (*n* = 3) of the patients [51]. In the over‐the‐top technique described by Di Sarsina et al. [40], none of the patients developed a valgus knee after ACLR with LET fixed proximal to the femoral physis [40]. The ‘tenodesis effect’ is therefore yet to be determined in future studies.

Biomechanical studies showed different results on stability and overconstraint after LET [21, 43, 47]. Evidence of biomechanical outcomes is low, due to small number of cadaveric knees. Probably due to ethical considerations, no studies were performed in skeletally immatures. Sena et al. ITB over‐the‐top ACLR with LET resulted in most studies in good stability and the addition of LET in the ITB over‐the‐top procedure improved stability [43]. There are concerns regarding overconstraint, as it may lead to an increase in tibiofemoral contact pressures resulting in accelerated degeneration [34]. Overconstraint in the ITB over‐the‐top technique is reported in two studies [43, 47]. This might be due to the degree of external rotation of the tibia during femoral fixation of the LET [17, 47]. Trentacosta et al. [47] fixed the ITB to the femur with the foot in neutral rotation, whilst Kennedy et al. [21] and Sena et al. [43] held the foot in 15° of external rotation. Thus, current biomechanical studies do not show a superior ACLR technique regarding knee kinematics, but overconstraint of the ITB over‐the‐top ACLR technique is a risk that is likely reduced by not placing the foot in external rotation while fixating the LET to the femur [47].

The addition of an ALLR in an all‐epiphyseal ACLR did not improve rotational stability in the cadaveric knees [47]. This seemed to be in contrast to the LET in the ITB over‐the‐top procedure, in which the stability of the knee was similar in ALL intact and ALL deficient states [47]. The LET of the ITB over‐the‐top ACLR has therefore a stabilising effect on the knee [47]. Clinical outcomes of hybrid ACLR + ALLR versus hybrid ACLR + LET showed no differences in complications [14]. There are currently no other studies investigating clinical outcomes after ALLR in skeletally immature patients. Future studies have to determine whether LET or ALLR are best to use as anterolateral augmentation procedure in the skeletally immature.

### Limitations

Limitations of this scoping review are due to the design of the included studies, which are technical notes, cadaveric studies, case series or retrospective cohort studies. There are currently no randomised controlled trials on anterolateral augmentation procedures in skeletally immature patients. There is not only a great variety in techniques of the ACLR and anterolateral augmentation procedures but also in the evaluation of outcomes such as growth disturbances, which makes comparing outcomes between studies difficult.

## CONCLUSION

This scoping review is the first to show results and surgical techniques of LET and ALLR with ACLR in skeletally immatures. There are currently two studies evaluating ACLR + LET versus isolated ACLR with promising results. There is little evidence on ALLR in skeletally immature patients and the effects of anterolateral augmentation on growth and overconstraint.

## AUTHOR CONTRIBUTIONS

Martijn Dietvorst was involved in conception and design of the study, data extraction, analyses and interpretation and drafting the manuscript. Stéphanie Verhagen contributed to data extraction, interpretation of the data and revision of the manuscript. Marieke C. van der Steen was involved in conception and design of the study, data analysis and interpretation and revision of the manuscript. Florens Q. M. P. van Douveren performed revision of the manuscript. Rob P. A. Janssen was involved conception and design of the study, data interpretation and revision of the manuscript. All authors read and approved the final manuscript.

## CONFLICT OF INTEREST STATEMENT

The authors declare no conflict of interest.

## ETHICS STATEMENT

Not applicable.

## Supporting information

Supporting information.

Supporting information.

Supporting information.

Supporting information.

## Data Availability

All data generated or analysed during this study are included in this published article (and its Supporting Information files).
