# Supplementary material for: Anterolateral augmentation procedures during anterior cruciate ligament reconstructions in skeletally immature patients: Scoping review of surgical techniques and outcomes
Source: J Exp Orthop. 2024 Mar 6;11(1):e12012. doi: 10.1002/jeo2.12012 (PMC10915482; doi:10.1002/jeo2.12012)

# Appendix 1 - Search strategy

***Update 22-12-2022***

| **EMBASE.com** | **PUBMED** | **COCHRANE** | **Total** | **Duplicates removed** |
| --- | --- | --- | --- | --- |
| 85 | 41 | 15 | 141 | 198 |

***5-10-2022***

| **EMBASE.com** | **PUBMED** | **COCHRANE** | **Total** | **Duplicates removed** |
| --- | --- | --- | --- | --- |
| 120 | 84 | 88 | 292 | 135 |

| **EMBASE.com** | **PUBMED** | **COCHRANE** | **Total** | **Duplicates removed** |
| --- | --- | --- | --- | --- |
| 971 | 811 | 80 | 1862 | 1152 |

### Pubmed 22-12-2022

Bovenkant formulier

| **Search** | **Query** | **Results** |
| --- | --- | --- |
| #4 | Search: **#3 AND ("2022/09/01"[Date - Entrez] : "3000"[Date - Entrez])** | 41 |
| #3 | Search: **#1 AND #2** | 909 |
| #2 | Search: **"Lateral extra-articular*"[tiab] OR LET[tiab] OR "anterolateral ligament*"[tiab] OR lemaire[tiab] OR ALL[tiab] OR "over-the-top"[tiab] OR "extra epiphyseal"[tiab] OR ("intra-articular"[tiab] AND "extra-articular"[tiab])** | 57,880 |
| #1 | Search: **Anterior Cruciate Ligament[Mesh] OR Anterior Cruciate Ligament Reconstruction[Mesh] OR Anterior Cruciate Ligament Injuries[Mesh] OR "anterior cruciate ligament*"[tiab] OR "anterior crucial ligament*"[tiab] OR "cranial cruciate ligament*"[tiab] OR ACL[tiab] OR ACLR[tiab] OR transphyseal[tiab] OR "all-epiphyseal"[tiab] OR "partial epiphyseal"[tiab] OR "physeal sparing"[tiab]** | 31,499 |

### Pubmed 5-10-2022

Onderkant formulier

| **Search** | **Query** | **Results** |
| --- | --- | --- |
| #4 | Search: **#3 AND ("2022"[Date - Entrez] : "3000"[Date - Entrez])** Sort by: **Publication Date** | [84](https://pubmed.ncbi.nlm.nih.gov/?term=%233+AND+%28%222022%22%5BDate+-+Entrez%5D+%3A+%223000%22%5BDate+-+Entrez%5D%29&sort=pubdate) |
| #3 | Search: **#1 AND #2** Sort by: **Publication Date** | [883](https://pubmed.ncbi.nlm.nih.gov/?term=%231+AND+%232&sort=pubdate) |
| #2 | Search: **"Lateral extra-articular*"[tiab] OR LET[tiab] OR "anterolateral ligament*"[tiab] OR lemaire[tiab] OR ALL[tiab] OR "over-the-top"[tiab] OR "extra epiphyseal"[tiab] OR ("intra-articular"[tiab] AND "extra-articular"[tiab])** Sort by: **Publication Date** | [57,184](https://pubmed.ncbi.nlm.nih.gov/?term=%22Lateral+extra-articular%2A%22%5Btiab%5D+OR+LET%5Btiab%5D+OR+%22anterolateral+ligament%2A%22%5Btiab%5D+OR+lemaire%5Btiab%5D+OR+ALL%5Btiab%5D+OR+%22over-the-top%22%5Btiab%5D+OR+%22extra+epiphyseal%22%5Btiab%5D+OR+%28%22intra-articular%22%5Btiab%5D+AND+%22extra-articular%22%5Btiab%5D%29&sort=pubdate) |
| #1 | Search: **Anterior Cruciate Ligament[Mesh] OR Anterior Cruciate Ligament Reconstruction[Mesh] OR Anterior Cruciate Ligament Injuries[Mesh] OR "anterior cruciate ligament*"[tiab] OR "anterior crucial ligament*"[tiab] OR "cranial cruciate ligament*"[tiab] OR ACL[tiab] OR ACLR[tiab] OR transphyseal[tiab] OR "all-epiphyseal"[tiab] OR "partial epiphyseal"[tiab] OR "physeal sparing"[tiab]** Sort by: **Publication Date** | [31,049](https://pubmed.ncbi.nlm.nih.gov/?term=Anterior+Cruciate+Ligament%5BMesh%5D+OR+Anterior+Cruciate+Ligament+Reconstruction%5BMesh%5D+OR+Anterior+Cruciate+Ligament+Injuries%5BMesh%5D+OR+%22anterior+cruciate+ligament%2A%22%5Btiab%5D+OR+%22anterior+crucial+ligament%2A%22%5Btiab%5D+OR+%22cranial+cruciate+ligament%2A%22%5Btiab%5D+OR+ACL%5Btiab%5D+OR+ACLR%5Btiab%5D+OR+transphyseal%5Btiab%5D+OR+%22all-epiphyseal%22%5Btiab%5D+OR+%22partial+epiphyseal%22%5Btiab%5D+OR+%22physeal+sparing%22%5Btiab%5D+&sort=pubdate) |

### Search strategy Elsevier/Embase.com 22-12-2022

| No. | Query | Results |
| --- | --- | --- |
| #4 | #3 AND [01-09-2022]/sd | 85 |
| #3 | #1 AND #2 | 1117 |
| #2 | 'lateral extra articular':ti,ab,kw OR let:ti,ab,kw OR 'anterolateral ligament*':ti,ab,kw OR 'all reconstruction*':ti,ab,kw OR lemaire:ti,ab,kw OR 'over-the-top':ti,ab,kw OR 'extra epiphyseal':ti,ab,kw OR (('intra-articular' NEAR/10 'extra-articular'):ti,ab,kw) | 32707 |
| #1 | 'anterior cruciate ligament'/exp OR 'anterior cruciate ligament reconstruction'/exp OR 'anterior cruciate ligament injury'/exp OR 'anterior cruciate ligament*':ti,ab,kw OR 'anterior cruciate knee ligament*':ti,ab,kw OR acl:ti,ab,kw OR 'anterior crucial ligament*':ti,ab,kw OR 'cranial cruciate ligament*':ti,ab,kw OR transphyseal:ti,ab,kw OR 'all-epiphyseal':ti,ab,kw OR 'partial epiphyseal':ti,ab,kw OR 'physeal sparing*':ti,ab,kw | 39385 |

### Search strategy Elsevier/Embase.com 05-10-2022

| **No.** | **Query** | **Results** |
| --- | --- | --- |
| #4 | #3 AND [01-01-2022]/sd | 120 |
| #3 | #1 AND #2 | 1067 |
| #2 | 'lateral extra articular':ti,ab,kw OR let:ti,ab,kw OR 'anterolateral ligament*':ti,ab,kw OR 'all reconstruction*':ti,ab,kw OR lemaire:ti,ab,kw OR 'over-the-top':ti,ab,kw OR 'extra epiphyseal':ti,ab,kw OR (('intra-articular' NEAR/10 'extra-articular'):ti,ab,kw) | 32707 |
| #1 | 'anterior cruciate ligament'/exp OR 'anterior cruciate ligament reconstruction'/exp OR 'anterior cruciate ligament injury'/exp OR 'anterior cruciate ligament*':ti,ab,kw OR 'anterior cruciate knee ligament*':ti,ab,kw OR acl:ti,ab,kw OR 'anterior crucial ligament*':ti,ab,kw OR 'cranial cruciate ligament*':ti,ab,kw OR transphyseal:ti,ab,kw OR 'all-epiphyseal':ti,ab,kw OR 'partial epiphyseal':ti,ab,kw OR 'physeal sparing*':ti,ab,kw | 39385 |

### Search strategy Wiley/Cochrane Library 22-12-2022

Date Run: 22/12/2022 10:18:32

| **ID** | **Search** | **Hits** |
| --- | --- | --- |
| #8 | #7 with Cochrane Library publication date Between Sep 2022 and Dec 2022 | 15 |
| #7 | #1 AND #6 | 100 |
| #6 | #2 OR #5 | 1409 |
| #5 | #3 AND #4 | 82 |
| #4 | ("extra-articular"):ti,ab,kw | 457 |
| #3 | ("intra-articular"):ti,ab,kw | 4326 |
| #2 | (“lateral extra articular” OR let OR “anterolateral ligament*” OR “all reconstruction*” OR lemaire OR “over-the-top” OR “extra epiphyseal”):ti,ab,kw | 1332 |
| #1 | ("anterior cruciate ligament*" OR "anterior cruciate knee ligament*" OR acl OR "anterior crucial ligament*" OR "cranial cruciate ligament*" OR transphyseal OR "all-epiphyseal" OR "partial epiphyseal" OR "physeal sparing*"):ti,ab,kw | 3623 |


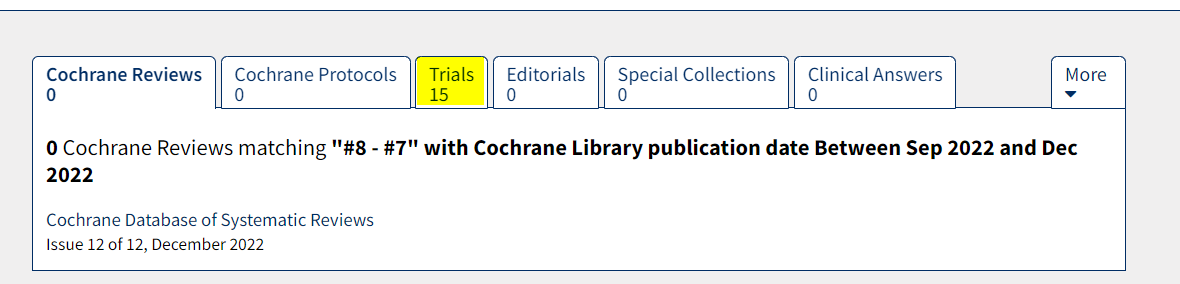


### Search strategy Wiley/Cochrane Library 05-10-2022

| **ID** | **Search** | **Hits** |
| --- | --- | --- |
| **#1** | ("anterior cruciate ligament*" OR "anterior cruciate knee ligament*" OR acl OR "anterior crucial ligament*" OR "cranial cruciate ligament*" OR transphyseal OR "all-epiphyseal" OR "partial epiphyseal" OR "physeal sparing*"):ti,ab,kw | **3510** |
| **#2** | (“lateral extra articular” OR let OR “anterolateral ligament*” OR “all reconstruction*” OR lemaire OR “over-the-top” OR “extra epiphyseal”) | **1729** |
| **#3** | "intra-articular":ti,ab,kw | **4248** |
| **#4** | "extra-articular":ti,ab,kw | **435** |
| **#5** | #3 AND #4 | **78** |
| **#6** | #2 OR #5 | **1803** |
| **#7** | #1 AND #6 | **88** |


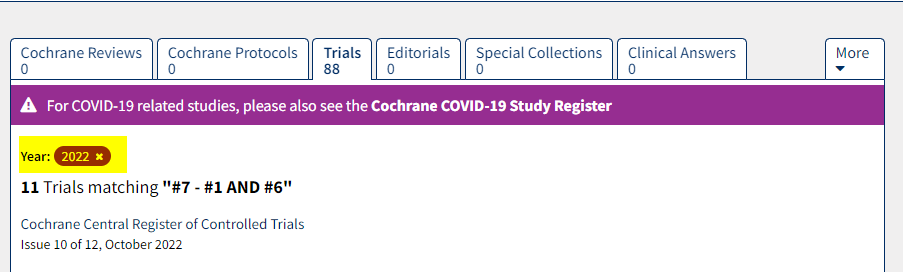

Supplement: Supplementary file 1 — Supporting information. [file JEO2-11-e12012-s003.docx]
