# Supplementary material for: Anterolateral augmentation procedures during anterior cruciate ligament reconstructions in skeletally immature patients: Scoping review of surgical techniques and outcomes
Source: J Exp Orthop. 2024 Mar 6;11(1):e12012. doi: 10.1002/jeo2.12012 (PMC10915482; doi:10.1002/jeo2.12012)
Supplement: Supplementary file 2 — Supporting information. [file JEO2-11-e12012-s004.docx]

# Appendix 2 – Study characteristics

| **Study** | **Year** | **Design** | **Anterolateral augmentation procedure** | **Indications** | **Number** | **Mean age, years** | **Sex,**  **n (%)** | **Outcomes** |
| --- | --- | --- | --- | --- | --- | --- | --- | --- |
| Dean et al.[8] | 2020 | Technical note | LET | Revision ACL  Primary ACL reconstruction in case of:  - High-grade pivot shift  - Generalized ligamentous hyperlaxity  - Increased posterior tibial slope (>12°)  Secondary indications:  - Chronic ACL insufficiency  - Meniscus insufficiency  - Depression of lateral femoral condyle (notch sign)  - High level athletes in pivoting sports | n.a. | n.a. | n.a. | n.a. |
| Feller et al.[12] | 2021 | Case series | LET | In case of high risk of graft rupture, at least two of following risk factors:  - Younger than 20 years of age at time of surgery  - Previous contralateral ACL reconstruction  - Positive family history of ACL rupture  - Generalized ligamentous laxity (Beighton ≥4)  - Grade 3 pivot shift  - Desire to return to a pivoting sports  - Elite or professional status | 25  2 with open growth plates | 18.5 (range 13.8 - 28.7)  Age of patients with open growth plates unknown | Female  4 (16)  Male  21 (84)  Open growth plates: unknown | - IKDC  - KOOS  - ACL-RSI  - Marx Activity Scale  - SANE score  - Range of motion  - KT-1000 arthrometer  - Single hop and triple crossover hop (LSI)  - Further injuries and surgeries to the knee |
| Foissey et al. [14] | 2022 | Case series | Two techniques:  ALLR and LET | Any child with growth potential (bone age younger than 13.5 years old for girls and younger than 15.5 years old for boys) who underwent a surgical technique combining ACL and lateral extra-articular procedure. | 39 patients (40 knees) | 13.8 (+/- 1.4) | 8 (20%) females  32 (80%) males | Clinical examination  PROMs   - Tegner - Lysholm - Pedi-IKDC   Complications  Growth disturbances clinically evaluated and confirmed with imaging |
| Kennedy et al.[21] | 2011 | Cadaveric laboratory study | LET | n.a. | n.a. | n.a. | n.a. | Displacement during stress |
| Kocher et al.[22] | 2006 | Technical note | LET | A complete midsubstance tear  of the anterior cruciate ligament  in a prepubescent child (Tanner  stage 1 or 2) for whom nonoperative  treatment consisting of rehabilitation,  bracing, and activity  restriction has failed.  These patients have symptoms  related to knee pivoting or further  meniscal or chondral injury related to instability. | - | - | - | - |
| Kocher et al.[23] | 2018 | Retro-spective cohort | LET | Children who were prepubescent  (Tanner stage 1 or 2) by maturity assessment at the  time of surgery and who underwent physeal-sparing, combined  intra- and extra-articular reconstruction of the ACLwith  IT band autograft between October 1989 and October 2012. | 237 patients  (240 knees) | 11.2  (SD 1.7) (range 5.7 - 15.6) | Female  33 (14)  Male  204 (86) | Medical records and imaging studies  were reviewed for demographic and clinical data, including  injury mechanism, presentation, concurrent injuries and/or  procedures, physical examination findings, and complications  PROMs   - Pedi-IKDC - Lysholm - Tegner |
| Lanzetti et al.[24] | 2020 | Retrospective cohort | LET | Patients with open femoral and tibial physis who underwent ACLR with the Over the Top technique and a long-term follow-up. | 42 patients | 12.5 years (11-14) | Females 12  Males 30 | Clinical examination  Graft failures  Return to sports  Growth disturbances (full length radiographs)  PROMs   - Pedi-IKDC - Tegner |
| Leyes-Vence et al.[25] | 2019 | Technical note | LET | During primary ACL:  - Grade 3 pivot shift test  - Segond fracture  - Chronic ACL rupture  - High sport level  - Pivoting sports  - <25 years old  - Preoperative side-to-side laxity of 7mm  - Lateral femoral notch sign  - In case of skeletally immaturity:  > valgus alignment  > recurvatum alignment  > generalized hyperlaxity  ACL revision | n.a. | n.a. | n.a. | n.a. |
| Monaco et al.[31] | 2022 | Retro-spective cohort study | LET | Only patients with ≥1 of the following additional risk  factors for a graft rupture (pivot-shift grade 2 or 3, high  level of sporting activity defined as Tegner activity score  ≥7, participation in pivoting sports, and Segond fractures). | 111 total  71 under-went ACLR+LET | 16.1 (range, 13.0-17.6)  Tanner  3/4: 35 (49%)  5: 36 (51) | Female  27 (38)  Male  44 (62) | Clinical examination  KT-1000 arthrometer  Graft failure  Reoperations  PROMs   - KOOS - IKDC - Tegner |
| Morin et al.[32] | 2022 | Technical note | ALL | n.a. | n.a. | n.a. | n.a. | n.a. |
| Patel et al.[37] | 2020 | Technical note | ALL | During primary ACL reconstruction in case of:  - knee hyperextension >10  - Beighton score >4  - Grade 2 pivot shift or higher  - Age, sex and sports are taken into consideration  Revision ACL reconstruction | n.a. | n.a. | n.a. | n.a. |
| Perelli et al.[38] | 2022 | Multicentre cohort study | LET | The included patients were skeletally immature indi- viduals with a maximum bone age of 16 years in boys and 14 years in girls. There had to be evidence of both tibial and femoral open epiphyseal growth plates on magnetic resonance imaging (MRI) scans and a diagnosis of a pri- mary ACL rupture. In addition, only patients willing to return to sports activities after the rehabilitation process were included. Only patients who had undergone ACL reconstruction with an autologous hamstring graft were considered for the enrollment. | 66 patients  32 undergone ACLR+LET | 13.8 (+/-1.4) years  Bone age 14.1 (+/-1.0) | Female 12  Male 20 | Graft failure  Objective knee stability with KT-1000 arthrometer and KiRA  Return to sports  PROMs   - HSS PEdi-FABS - Pedi-IKDC   Complications |
| Di Sarsina et al.[42] | 2018 | Case series | LET | Age under 14 years and Tanner stage  between I and IV at the time of surgery  Clinical and/or  radiographic evidence for ACL rupture  Growth plate of the proximal tibia still open at the time of the surgery  Regular participation in sport activities  Asymptomatic, stable, and functional contralateral knee  BMI < 30 kg/  m^2^  Minimum follow-up of 30 months | 20 | 12.3 (SD 1.7) | Female 10 (50)  Male  10 (50) | Physical examination  Re-injuries  KT-1000 arthrometer and Rolimeter  Knee and long leg radiographs  PROMs   - KOOS - IKDC - Lysholm - Tegner |
| Schlichte et al.[43] | 2020 | Technical note | LET | During primary ACL reconstruction in case of:  - 13-15 years of age  - Participating in high-risk competitive sports requiring cutting, pivoting and jumping  - Grade 3 pivot shift  - Anterior translation >7mm side-to-side difference  - Lateral tibial slope >7  - Recurvatum >10  - Notch width index <0.22  - Large marrow edema patterns involving lateral tibial plateau and lateral femoral condyle  - Hyperlaxity Beighton >6  - Chronic ACL insufficiency  - Contralateral ACL reconstruction  Revision ACL reconstruction | n.a. | n.a. | n.a. | n.a. |
| Sena et al.[44] | 2013 | Cadaveric laboratory study | LET | n.a. | n.a. | n.a. | n.a. | Kinematics and load conditions |
| Shamrock et al.[45] | 2022 | Retro-spective cohort | LET | Patient with ACL injury and with open tibial and femoral physes. | 12 | 12 (SD 1.2; range 10-16) | Female 1 (8)  Male 11 (92) | Clinical examination  Graft failure  Complications and subsequent injuries  Return to sport |
| Trentacosta et al.[47] | 2017 | Cadaveric laboratory study | ALL | n.a. | n.a. | n.a. | n.a. | Displacement during stress |
| Willimon et al.[50] | 2015 | Case series | LET | Micheli reconstruction was recommended  for patients with ≥3 years of growth remaining  and was an option for slightly more mature patients whose  families were particularly concerned about growth disturbances. | 21 patients  22 knees | 11.8 (range, 9.9-14.0) | Female  0 (0)  Male  21 (100) | Clinical examination  Radiographic examination  PROMs   - Lysholm knee scale - Tegner activity scale - Pedi-IKDC |
| Wilson et al.[51] | 2019 | Case series | LET | Not mentioned  Inclusion criteria:  - skeletally immature (<3 years remaining growth based on bone age Greulich and Pyle)  - Minimum of 2 years follow-up  - Transphyseal and ITB ACL reconstruction | 57 | Chronological age 13.0  Bone age  13.9 | Female 21 (37)  Male  36 (63) | - Radiographic outcomes including leg length and alignment and joint degeneration  - Secondary injuries  - Surgery to RTP clearance  - Return to sport (%)  - Pedi-IKDC  - HSS Pedi-FABS  - Graft failure  - Surgery to graft failure |
