# Supplementary material for: Anterolateral augmentation procedures during anterior cruciate ligament reconstructions in skeletally immature patients: Scoping review of surgical techniques and outcomes
Source: J Exp Orthop. 2024 Mar 6;11(1):e12012. doi: 10.1002/jeo2.12012 (PMC10915482; doi:10.1002/jeo2.12012)
Supplement: Supplementary file 3 — Supporting information. [file JEO2-11-e12012-s002.docx]

# Appendix 3 – Lateral extra-articular tenodesis techniques

| **Study** | **LET** | | | | **ACL reconstruction** | | | |
| --- | --- | --- | --- | --- | --- | --- | --- | --- |
|  | **Technique based** | **Graft** | **Fixation position** | **Fixation method** | **Technique** | **Graft type** | **Fixation** | **Diameter (mm)** |
| Dean et al.[8] | Lemaire | ITB | Femoral: adjacent to the distal Kaplan fiber attachment; distal to growth plate or proximal at anatomic origin of ALL  Tibial: not detached Gerdy tubercle  Knee at 30 degrees of flexion and neutral rotation | Femoral: suture anchor  Tibial: original insertion  ITB closure with sutures | Transphyseal ACL reconstruction  (help of Gore smoother) | Hamstring tendon autograft | Femur: button  Tibia: screw and washer | - |
| Feller et al.[12] | Ellison | ITB | Femoral: not detached, preserving Kaplan fibers  Tibial: bony trough in Gerdy tubercle | Femoral: not detached  Tibial: bone anchor in bony trough at Gerdy tubercle and distal end of strip is reattached with sutures with reinforcement sutures; ITB defect usually left open | Not specified | Hamstring tendon autograft in case of open growth plates | Whipstitch tied to a fixation post | - |
| Foissey et al.[14] | Lemaire | ITB | Femoral: fixed on the wires of the ACL button  Tibial: not detached from Gerry's tubercle  Tensioning position not mentioned | Femoral: fixed on ACL button with wires  Tibial: not detached | Hybrid (femoral all-epiphyseal outside-in with entry point posterior and proximal to the lateral epicondyle and tibial transphyseal) | Hamstring tendons | Femoral: cortical button  Tibial: not detached from pes anserinus | ≤9 |
| Kennedy et al.[21] | Modified MacIntosh and Darby / Micheli | ITB | Femoral: lateral condyle  Tibial: not detached Gerdy tubercle  Knee at 90 degrees of flexion and 15 degrees of external rotation | Femoral: staple  Tibial: not detached | Over-the-top | ITB | Femoral: staple at lateral condyle  Tibial: distal and medial to tibial tubercle with staple and post | - |
| Kocher et al.[22, 23] | Modified MacIntosh and Darby / Micheli | ITB | Femoral: lateral femoral condyle at insertion of lateral intermuscular septum  Tibial: not detached Gerdy tubercle  Knee at 90 degrees of flexion and 15 degrees of external rotation | Femoral: mattress sutures  Tibial: not detached | Over-the-top | ITB | Femoral: lateral femoral condyle at insertion of lateral intermuscular septum with mattress sutures  Tibial: proximal tibial medial metaphyseal  Cortex. The graft is sutured  to the periosteum at the  rough margins of the trough  with mattress sutures | - |
| Lanzetti et al.[24] | Modified Marcacci | HS | Femoral: fixed in the over-the-top position  Tibia: fixed at Gerdy’s tubercle  Tensioning position not mentioned | Femoral: two staples  Tibia: one staple | Over-the-top | Hamstring tendons | Femoral: fixed in the over-the-top position  Tibia: not detached from pes anserinus | - |
| Leyes-Vence et al.[25] | Lemaire | ITB | Femoral: just proximal and posterior from lateral epicondyle  Tibial: not detached from Gerdy tubercle  Knee at 30 degrees of flexion and exorotation | Femoral: attachment at ACL reconstruction graft button  Tibial: not detached from Gerdy tubercle | Partial epiphyseal | Hamstring tendon autograft | Femur and tibia button | ≥8 |
| Monaco et al.[31] | Arnold-Cooker modification of MacIntosh | ITB | Femoral: no fixation  Tibial: attachment at Gerdy intact; ITB is sutured on itself at the level of Gerdy and LCL  Knee in 90 degrees of flexion and held in external rotation | Femoral: -  Tibial: ITB suture with absorbable stitches on itself | Transphyseal | Quadrupled hamstring tendon autograft | Femur cortical suspensory device  Tibia interference screw | 8.3 (range 7-10) |
| Perelli et al.[38] | Lemaire | ITB | Femoral: 1 cm proximal to the origo of the ALL  Tibial: no detachment from Gerdy's tubercle  Tensioning et 30 degrees of flexion and neutral rotation | Femoral: intereference screw  Tibial not detached | Hybrid (all-epiphyseal femur and transphyseal tibia) | Quadrupled hamstring tendon autograft | Femoral: cortical suspension button  Tibial: interference screw | 8.3 (SD 1.1) |
| Di Sarsina et al.[42] | Marcacci | HS | Femoral: lateral femoral condylar-metaphyseal junction (proximal to the physis)  Tibial: Gerdy’s tubercle | Femoral: two staples  Tibial: staple | Over-the-top | Hamstring tendon autograft | Femoral: proximal to the physis at junction with two staples  Tibial: preserving insertion at pes anserinus | - |
| Schlichte et al.[43] | Lemaire | ITB | Femoral: just proximal and posterior from lateral epicondyle  Tibial: not detached from Gerdy tubercle  Knee at 30 degrees of flexion and neutral rotation | Femoral: all-suture anchor in femur; attachment of graft over LCL with non-absorbable suture on itself  Tibial: not detached from Gerdy tubercle | Not specified | Quadriceps tendon | Not specified | Not specified |
| Sena et al.[44] | Modified MacIntosh and Darby / Micheli | ITB | Femoral: lateral condyle  Tibial: not detached Gerdy tubercle  Knee at 90 degrees of flexion | Femoral: staple  Tibial: not detached | Over-the-top | ITB | Femoral: staple at lateral condyle  Tibial: distal and medial to tibial tubercle with staple and post | - |
| Shamrock et al.[45] | Modified MacIntosh | ITB | Femoral: approximately 1.5-3.0 cm proximal to the distal femoral  physis  Tibial: not detached from Gerdy tubercle  Knee at 15 degrees of flexion while applying posterior drawer | Femoral: fully threated cancellous screws and washer or cannulated cancellous screws with spiked washer  Tibial: not detached from Gerdy tubercle | Over-the-top and partial transphyseal | ITB | Femoral: approximately 1.5-3.0 cm proximal to the distal femoral  Physis with screw and washer  Tibial: staple or fully threated cancellous screws and washer distal to the tibial physis | - |
| Trentacosta et al.[47] | Modified MacIntosh and Darby / Micheli | ITB | Femoral: lateral femoral condyle at insertion of lateral intermuscular septum  Tibial: not detached Gerdy tubercle  Knee at 90 degrees of flexion and 15 degrees of external rotation | Femoral: mattress sutures  Tibial: not detached | Over-the-top | ITB | Femoral: lateral femoral condyle at insertion of lateral intermuscular septum with mattress sutures  Tibial: proximal tibial medial metaphyseal  Cortex. The graft is sutured  to the periosteum at the  rough margins of the trough  with mattress sutures | - |
| Willimon et al.[50] | Modified MacIntosh and Darby / Micheli | ITB | Femoral: lateral femoral condyle at insertion of lateral intermuscular septum  Tibial: not detached Gerdy tubercle | Femoral: sutures  Tibial: not detached | Over-the-top | ITB | Femoral: lateral femoral condyle with sutures  Tibial: secured to the tibia just distal and medial to the tibial tubercle using an anchor | - |
| Wilson et al.[51] | Modified MacIntosh | ITB | Femoral: lateral femoral condylar-metaphyseal junction  Tibial: not detached from Gerdy tubercle (intra-articular portion in tibial tunnel together with hamstring tendon autograft)  Knee in extension and neutral rotation | Femoral: No. 2 non-absorbable suture to periosteum  Tibial: not detached from Gerdy tubercle (intra-articulair portion interference screw) | Transphyseal + extraphyseal ACL reconstruction (over-the-top) | Hamstring tendon autograft + ITB | Femur: button  Tibia: interference screw | 7.9 (range 5-9.5) |
