# Supplementary material for: Anterolateral augmentation procedures during anterior cruciate ligament reconstructions in skeletally immature patients: Scoping review of surgical techniques and outcomes
Source: J Exp Orthop. 2024 Mar 6;11(1):e12012. doi: 10.1002/jeo2.12012 (PMC10915482; doi:10.1002/jeo2.12012)
Supplement: Supplementary file 4 — Supporting information. [file JEO2-11-e12012-s001.docx]

# Appendix 4 – Anterolateral ligament reconstruction techniques

| **Study** | **ALLR** | | | | **ACLR** | | | |
| --- | --- | --- | --- | --- | --- | --- | --- | --- |
|  | Type | Graft | Fixation position | Fixation method | Technique | Graft type | Fixation | Diameter (mm) |
| Foissey et al.[14] | ALLR | Gracilis tendon (double bundle) | Double tunnel:   - Anterior tunnel slightly posterior to Gerdy's tubercle - Posterior tunnel midway between Gerdy's tubercle and fibular head | Femoral: screw  Tibial: not specified | Hybrid (femoral all-epiphyseal outside-in with entry point posterior and proximal to the lateral epicondyle and tibial transphyseal) | Hamstring tendons  (3 strands ST and 1 strand G tendon) | Femoral: cortical screw  Tibial: not detached from pes anserinus | ≤9 |
| Morin et al.[32] | ALLR | Continuous ST and G graft | Femur: distal to the physis, proximal and posterior to the epicondyle  Tibia: two convergent tunnels at Gerdy tubercle and lateral to Gerdy tubercle | Femur: ACL traction wire  Tibia: no fixation | Partial epiphyseal | Hamstring tendon autograft | Femur: interference screw  Tibia: anchor | 8 – 9 |
| Patel et al.[37] | ALLR + anterolateral capsular reinforcement | ITB | Anterolateral capsular reinforcement: not detached from Gerdy tubercle  Femoral: just proximal and posterior from lateral epicondyle  Tibial: midpoint between Gerdy tubercle and fibular head just proximal from physis  Knee in extension and neutral rotation during graft tensioning | Femoral: No. 0 non-absorbable suture stitch to periosteum (in 90 degrees knee flexion)  Tibial: No. 0 non-absorbable suture stitch to periosteum (in full knee extension) | All-epiphyseal ACL reconstruction | Quadriceps tendon autograft | Femur and tibia button | - |
| Trentacosta et al.[47] | ALLR | Posterior tibialis tendon allograft | Femoral: just proximal and posterior from lateral epicondyle  Tibial: midpoint between Gerdy tubercle and fibular head  Knee in extension | Femoral: 4.75mm anchor  Tibial: 7mm anchor | All-epiphyseal ACL reconstruction | Hamstring tendon autograft | Femur and tibia button | 9mm (range 8-9mm) |
